# Tuning the Structure of Poly(aspartic acid)s’ Self-Assemblies to Enhance Cellular Uptake

**DOI:** 10.3390/polym17172373

**Published:** 2025-08-31

**Authors:** Jimin Jeong, Junwoo Lim, Sungwoo Cho, Sa Ra Han, Suk Hyeon Hong, Jae Hyun Jeong

**Affiliations:** Department of Chemical Engineering, Soongsil University, 369, Sangdo-Ro, Dongjak-Gu, Seoul 06978, Republic of Korea; jamiej1123@soongsil.ac.kr (J.J.); jwlim@ncc.re.kr (J.L.); chosw1120@gmail.com (S.C.); srhan@ssu.ac.kr (S.R.H.); hsh4768@soongsil.ac.kr (S.H.H.)

**Keywords:** amphiphilic graft copolymers, morphological transition, degree of substitution, cellular uptake enhancement

## Abstract

Self-assembled nanoparticles formed with amphiphilic block or graft copolymers are being extensively studied for their use in a variety of biological and industrial applications, including targeted drug delivery. This study reports a novel strategy to tune the structure of self-assembled nanoparticles for enhancing the cellular uptake by varying the hydrophilic ratio of amphiphilic graft copolymers. We synthesized poly(aspartic acid) (PAsp) substituted with octadecyl chains (C18) at varying degrees of substitution (DS), ranging from 4.5 to 37.5 mol%, which could form self-assemblies in an aqueous solution. As the DS increased, a morphological transition was observed—from spherical assemblies (DS 4.5 and 9.1) to rod-like (DS 19.0), vesicular (DS 25.7), and lamellar-like structures (DS 37.6). Further, Trans-Activator of Transcription (TAT) as the cell penetrating peptide to the synthesized amphiphilic graft copolymers leads to an enhanced cellular uptake of the biomimetic self-assembly. In particular, the lamellar-like self-assemblies resulted in a 1.3-fold increase of cellular uptake, as compared to the spherical self-assemblies, and a 3.6-fold increase, as compared to the vesicles. Therefore, tuning the structure of poly(aspartic acid)s’ self-assemblies was proven as an effective strategy to enhance the cellular uptake, while minimizing invasive cell damage. This new strategy to tune the morphologies of self-assemblies will serve to improve the cell penetrating activity for targeted drug delivery.

## 1. Introduction

Nano-sized self-assemblies derived from amphiphilic block and graft copolymers have attracted significant interest due to their structural versatility, biodegradability, and potential in biomedical applications such as targeted drug delivery [1,2]. These supramolecular structures form spontaneously in aqueous environments via hydrophobic interactions, typically resulting in core–shell architectures that can encapsulate therapeutic agents. Poly(amino acid)-based copolymers are particularly promising due to their protein-mimetic backbones, which confer excellent biocompatibility and enzymatic degradability [3,4]. These polymers, composed of peptide-like amide linkages, can be synthetically modified to incorporate diverse functional groups, making them highly versatile platforms for the design of smart nanomaterials [5]. A widely used approach to inducing amphiphilicity in poly(amino acid)s is grafting hydrophobic side chains onto the hydrophilic polymer backbone. Such amphiphilic graft copolymers spontaneously form self-assembled nanostructures in aqueous environments, where the hydrophobic side chains drive core formation and the hydrophilic backbone stabilizes the shell [6,7,8]. This architecture enables the efficient encapsulation of hydrophobic drugs, protecting them from premature degradation and facilitating controlled release in physiological conditions [9,10].

Depending on the balance between hydrophobic and hydrophilic components, these self-assemblies can exhibit a range of morphologies, including spherical micelles, cylindrical micelles (rods), vesicles, and lamellar aggregates [11]. Controlling these morphologies is critical, as they directly influence drug loading efficiency, release profiles, circulation time, and cellular uptake behavior. For instance, vesicles offer dual-compartment loading for hydrophilic and hydrophobic drugs, while rod-like particles often show prolonged blood circulation and enhanced tumor penetration compared to spherical particles [10,12]. Various approaches, including the use of stimuli-responsive blocks, pH-sensitive linkages, or co-assembly with functional additives, have been employed to modulate nanoparticle morphology for specific biomedical functions [13,14].

A key challenge in nanoparticle-based drug delivery lies in ensuring efficient cellular internalization without causing adverse cytotoxic effects. While nanoparticle size has traditionally been considered a major determinant of uptake efficiency, growing evidence suggests that shape and surface characteristics also play critical roles in endocytic pathways [15]. In this context, cell-penetrating peptides (CPPs) have emerged as powerful tools to facilitate intracellular delivery of therapeutic cargoes. Among these, the Trans-Activator of Transcription (TAT) peptide, derived from HIV-1, has demonstrated robust cellular uptake via both direct penetration and endocytosis [16,17]. When conjugated to nanocarriers, CPPs can significantly enhance internalization, especially in cases where morphology or surface properties might otherwise limit cellular entry.

In this study, we aimed to fine-tune the morphology of amphiphilic graft copolymers based on poly(aspartic acid) (PAsp), denoted as PAsp-g-C18, by systematically varying the degree of substitution (DS) with hydrophobic octadecyl chains. Through this structural modulation, we induced morphological transitions—from spherical to rod-like, vesicular, and lamellar-like self-assemblies—under aqueous conditions. To evaluate their potential in intracellular delivery, the self-assemblies were further functionalized with the TAT peptide. We then investigated how differences in nanostructure morphology affect cellular uptake efficiency. Notably, the lamellar-like self-assemblies exhibited approximately 1.3-fold higher cellular uptake than the spherical counterparts and about 3.6-fold higher uptake compared to the vesicular structures. Our results reveal a strong correlation between morphology and cellular internalization, providing design insights for the development of next-generation polymeric drug carriers.

## 2. Materials and Methods

### 2.1. Synthesis of Poly(aspartic acid) Substituted with Octadecyl Chains

The precursor polymer, poly(succinimide) (PSI), was synthesized via acid-catalyzed thermal polycondensation of L-aspartic acid, as previously reported [3]. Briefly, L-aspartic acid (25 g, 0.188 mol) and phosphoric acid (9.4 mmol, Fisher Scientific, Waltham, MA, USA) as the catalyst were suspended in a mixed solvent of mesitylene and sulfolane (7:3, *w*/*w*; total 125 g). The mixture was refluxed at 170 °C under nitrogen purging, and the water produced during the reaction was continuously removed using a Dean–Stark trap coupled with a reflux condenser. After 12 h of reaction, the mixture was precipitated in excess methanol and washed repeatedly with deionized (DI) water until neutral pH was achieved, removing residual acid catalyst. The resulting precipitate was further washed with ethyl ether and dried at room temperature. The chemical structure of the obtained PSI was confirmed by ^1^H-NMR spectroscopy (400 MHz, AVANCE III, Bruker, Billerica, MA, USA) [18].

For hydrophobic modification, PSI (0.97 g) was dissolved in anhydrous DMF, and octadecylamine (C18) was separately dissolved in DMF at 70 °C. The C18 solution was then added dropwise to the PSI solution, and the reaction mixture was stirred at 70 °C for 24 h under a nitrogen atmosphere to induce aminolysis. After completion, the mixture was cooled to room temperature and precipitated in excess ethyl ether. The precipitate was collected by filtration and dried at room temperature. The degree of substitution (DS) of octadecylamine was controlled by varying the feed molar ratio (5, 10, 20, 40, and 60 mol%), and the resulting products were designated as PSI-g-C18-1 through PSI-g-C18-5, respectively. The chemical structure of the amphiphilic copolymers was confirmed by ^1^H NMR spectroscopy in DMSO-d_6_. Subsequently, 1 N NaOH was added to the PSI-g-C18 solution to hydrolyze the remaining succinimide units, and the mixture was stirred at room temperature for 3 h. The resulting solution was dialyzed for 3 days (MWCO = 8000–12,000 Da) against DI water to remove residual NaOH, followed by lyophilization. The final products were designated as PAsp-g-C18-1, PAsp-g-C18-2, PAsp-g-C18-3, PAsp-g-C18-4, and PAsp-g-C18-5, corresponding to increasing DS values.

### 2.2. Preparation and Characterization of PAsp-g-Alkyl Chain Self-Assemblies

Self-assemblies of PAsp-g-C18 copolymers with low degrees of substitution (PAsp-g-C18-1, PAsp-g-C18-2, and PAsp-g-C18-3) were prepared by dispersing 0.1 g of each copolymer in 10 mL of deionized (DI) water, followed by vortex mixing to form a milky suspension. The suspension was then sonicated using a bath-type sonicator (Powersonic 410) at room temperature for 10 min, resulting in clear solutions and facilitating the self-assembly of the graft copolymers. For copolymers with high degrees of substitution (PAsp-g-C18-4 and PAsp-g-C18-5), 0.1 g of each sample was dissolved in 1 mL of DMSO, a solvent suitable for both the PSI backbone and octadecylamine side chains. The DMSO solution was added dropwise into 10 mL of DI water under stirring to induce self-assembly. The resulting mixtures were dialyzed against DI water for 3 days using dialysis membranes (MWCO = 8000–12,000 Da) to remove residual DMSO. All self-assembled polymer solutions were filtered through 0.45 μm syringe filters (Whatman) to remove dust and aggregates prior to characterization.

The hydrodynamic size of the self-assembled nanoparticles was measured using dynamic light scattering (DLS; ZS90, Malvern Instruments Ltd., Worcestershire, UK) at room temperature. Measurements were conducted at a fixed scattering angle of 90°, using 1 mL of each polymer solution placed in disposable cuvettes. Each sample was measured in triplicate to ensure reproducibility. The critical aggregation concentration (CAC) of each sample was determined using the fluorescence excitation method with pyrene as a hydrophobic probe. Polymer solutions were prepared at concentrations ranging from 1 to 2.56 × 10^−6^ mg/mL. A small aliquot (4 μL) of pyrene stock solution in acetone was added to 2 mL of each polymer solution. After equilibration, the fluorescence spectra were recorded using a spectrofluorometer (QM40, PTI, Birmingham, NJ, USA), with excitation at 330 nm and emission spectra collected from 350 to 450 nm. The ratio of emission intensities at 385 nm (I_3_) and 373 nm (I_1_) was used to determine CAC, as a sharp increase in the I_3_/I_1_ value indicates the onset of micelle formation. The morphology of the self-assembled PAsp-g-C18 nanoparticles (DS 4.5–37.6 mol%) was examined using transmission electron microscopy (TEM; JEM-2010 HC, JEOL, Peabody, MA, USA) operated at an accelerating voltage of 120 kV. For sample preparation, each aqueous nanoparticle suspension was dropped onto a carbon-coated copper grid and allowed to adsorb for 10 min. The grid was then placed on a droplet of Uranyless^®^ negative stain on Parafilm for 1 min, followed by blotting with filter paper. The stained grids were air-dried for 2 h prior to imaging [19].

### 2.3. Synthesis of PAsp-g-TAT Grafted with Octadecyl Chains

Purified PSI (0.16 g) was dissolved in anhydrous DMF, and the HIV-1 Trans-Activator of Transcription (TAT) peptide (residues 47–57) was also dissolved in DMF at room temperature. The cell-penetrating peptide (CPP) conjugation reaction was initiated by adding the TAT solution dropwise to the PSI solution, followed by stirring at room temperature for 24 h under a nitrogen atmosphere. The DS of TAT was controlled by adjusting the feed molar ratio, and the resulting product was denoted as PSI-g-TAT. Next, hydrophobic modification was carried out by adding a DMF solution of octadecylamine to the PSI-g-TAT solution. The reaction was stirred at 70 °C for 24 h under nitrogen to promote aminolysis. After cooling to room temperature, the reaction mixture was precipitated in excess ethyl ether, and the solid product was collected by filtration and dried at ambient conditions. To hydrolyze the remaining succinimide rings and obtain the final product, the dried copolymer was treated with 1 N NaOH solution and stirred at room temperature for 3 h. The hydrolyzed product was then purified by dialysis (MWCO = 8000–12,000 Da) and finally lyophilized to yield PAsp-g-TAT-C18.

### 2.4. Synthesis of PHEA-g-NH_2_ Grafted with Octadecyl Chains

To synthesize octadecylamine-grafted poly(hydroxyethyl aspartamide) (PHEA-g-C18-NH_2_), PSI (0.97 g) was dissolved in anhydrous DMF. A DMF solution of octadecylamine was added to the PSI solution, and the mixture was stirred at 70 °C for 24 h in an oil bath under nitrogen atmosphere. After the reaction, the solution was cooled to room temperature, and ethanolamine was added to initiate ring-opening of the remaining succinimide units. The mixture was stirred for an additional 6 h at room temperature. Subsequently, the solution was added dropwise to an excess of ethylenediamine and stirred for another 24 h under nitrogen to complete the reaction. The final product was precipitated in ethyl ether, filtered, and dried under a fume hood. The overall synthetic scheme is illustrated in Figure S2.

### 2.5. Modular Assembly for Preparing Biomimetic Self-Assemblies

Stable biomimetic self-assemblies were prepared using a modular combination of PHEA-g-C18-NH_2_, PAsp-g-C18, and PAsp-g-C18-TAT, with FITC conjugation. The DS of octadecyl chains (C18) was fixed at 4.5 mol% in both PHEA-g-C18-NH_2_ and PAsp-g-C18-TAT. The polymers were dissolved in DMSO at a mass ratio of 1:8:1 and added dropwise into 10 mL of deionized water under gentle stirring. Separately, FITC dissolved in DMSO was added to the aqueous mixture and stirred for 3 h at room temperature [20]. The resulting reaction solution was dialyzed five times against deionized water using a dialysis membrane (MWCO = 8000–12,000 Da) to remove residual DMSO and unreacted FITC. The final nanoparticle dispersion was filtered through a 0.45 μm syringe filter (Whatman) to eliminate dust and aggregates. This procedure was repeated for each DS formulation, and the final stock solution concentration was adjusted to 0.5 mg/mL.

### 2.6. Human Dermal Fibroblast Culture and Cellular Uptake

Human dermal fibroblasts (HDFn; Gibco, Thermo Fisher Scientific, Waltham, MA, USA) were cultured in a growth medium composed of Human Fibroblast Expansion Medium (M106, Gibco, USA), supplemented with low-serum growth supplement (LSGS, Gibco, USA) and 1.0% (*v*/*v*) penicillin–streptomycin (P/S, Biowest, Nuaillé, France). Cells were maintained at 37 °C in a humidified atmosphere containing 5% CO_2_. For confocal microscopy, HDFn cells were seeded on sterile glass coverslips at a density of 3 × 10^4^ cells per coverslip and incubated overnight. After washing twice with PBS (pH 7.4), the cells were treated with FITC-labeled self-assemblies (diluted in culture medium, final concentration: 0.05 mg/mL) and incubated for 10 min or 2 h at 37 °C. Unbound nanoparticles were removed by washing twice with PBS, and cells were fixed with 4% paraformaldehyde. Imaging was performed using an LSM 900 confocal microscope (Carl Zeiss, Jena, Germany) with excitation at 490 nm and emission collected at 520 nm (green channel for FITC). For flow cytometry, HDFn cells were seeded in 6-well plates at a density of 1 × 10^6^ cells/well in 1 mL of medium and cultured until 80% confluency. After washing with PBS, the cells were incubated with either FITC-labeled or Nile Red-encapsulated nanoparticles (final concentration: 0.05 mg/mL) for 10 min or 2 h at 37 °C. Following incubation, cells were washed twice with PBS, detached using 0.25% trypsin, and centrifuged at 1000 rpm. The cell pellets were resuspended and fixed with 4% paraformaldehyde for 15 min at room temperature. After fixation, cells were washed by centrifugation and resuspended in 1 mL of 1× PBS. Flow cytometry analysis was performed using a BD LSRFortessa™ system (Becton Dickinson, San Jose, CA, USA), collecting 30,000 events per sample. FITC was excited using a 488 nm laser, and green fluorescence was detected using a 525 nm band-pass filter. Data were analyzed using FlowJo software (version 10, FlowJo LLC, Ashland, OR, USA).

## 3. Results and Discussions

### 3.1. Synthesis of Poly(aspartic acid)s Grafted with Alkyl Chains

**Figure 1:**
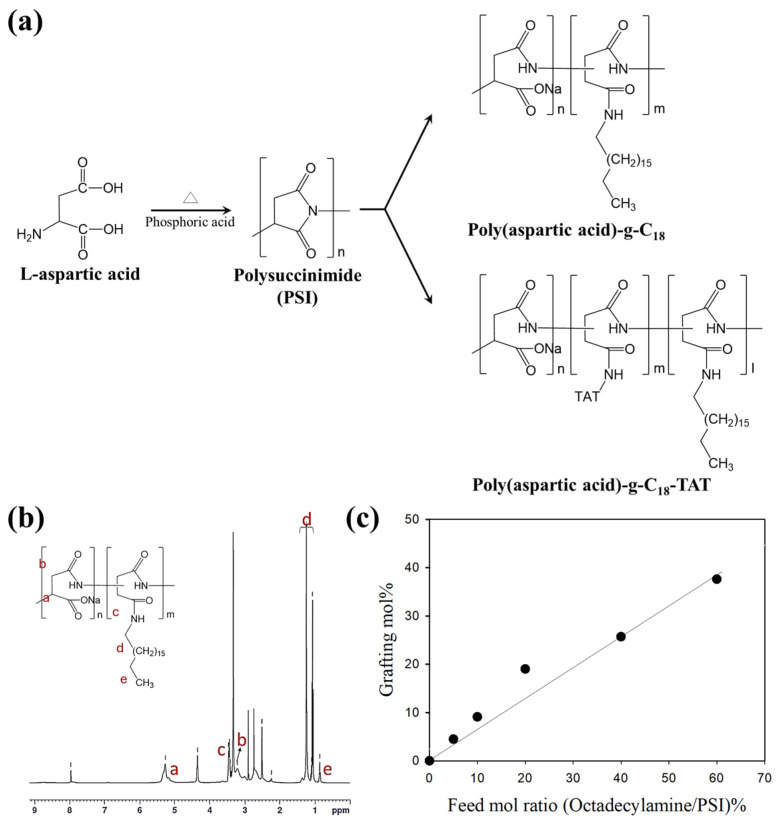
Synthetic route and characterization of poly(aspartic acid) (PAsp) graft copolymers with controlled substitution of octadecyl chains (C18). (**a**) Schematic illustration of the synthesis of amphiphilic PAsp grafted with octadecyl chains and TAT peptides. (**b**) ^1^H-NMR spectra of PAsp-g-C18 conjugates in D_2_O, confirming successful grafting of hydrophobic chains. (letters a–e correspond to the assigned proton signals in the molecular structure.) (**c**) Degree of substitution (mol%) of octadecyl chains to PSI as a function of the initial feed molar ratio (octadecylamine/succinimide units).

Amphiphilic graft copolymers composed of a hydrophilic poly(aspartic acid) (PAsp) backbone and hydrophobic octadecyl side chains were successfully synthesized to explore morphology-tuned self-assembly. The precursor, polysuccinimide (PSI), was prepared via acid-catalyzed thermal polycondensation of L-aspartic acid using phosphoric acid as a catalyst. Subsequently, octadecylamine and the cell-penetrating peptide TAT (Trans-Activator of Transcription) were sequentially conjugated to PSI in dimethylformamide (DMF) to produce PAsp-g-C18 and PAsp-g-TAT-C18, respectively (Figure 1a and Figure S1).

The chemical structure of PSI was confirmed by ^1^H-NMR spectroscopy, showing characteristic peaks at 2.7 ppm and 3.2 ppm (methylene protons of the succinimide ring), as well as at 5.3 ppm (methine proton of the polymer backbone) [21]. The DS of octadecyl chains (mol%) was determined by integrating the ^1^H-NMR spectra of PAsp-g-C18 (Figure 1b) and was tunable by varying the molar feed ratio of octadecylamine to PSI repeat units. As a result, a series of copolymers with DS values ranging from 4.5 to 37.6 mol% was obtained (Figure 1c and Figure S3). Also, FT-IR spectra of PAsp-g-C_18_ further supported the successful grafting, showing characteristic C–H stretching peaks (2850–3000 cm^−1^) that intensified with increasing DS and amide-related bands at 1550–1650 cm^−1^ corresponding to –NH bending and C=O stretching (Figure S4). Furthermore, TGA analysis revealed a typical two-step weight-loss behavior with negligible decomposition below 150 °C and major degradation occurring above 200 °C, with the onset of thermal decomposition decreasing slightly as DS increased (Figure S5).

These amphiphilic graft copolymers were designed to exhibit distinct self-assembled morphologies in aqueous media depending on the hydrophilic–hydrophobic balance, providing a modular platform for investigating morphology-dependent cellular uptake.

### 3.2. Characteristics of PAsp-g-C18 Self-Assembled Nanoparticles

**Figure 2:**
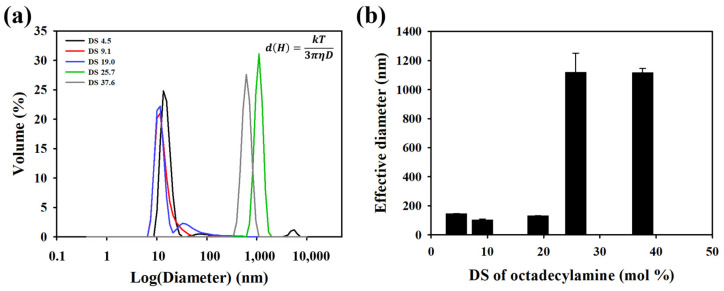
Size characterization of PAsp-g-C18 self-assembled nanoparticles with varying DS. (**a**) Size distribution profiles of PAsp-g-C18 self-assemblies in aqueous solution (0.5 mg/mL), measured by dynamic light scattering (DLS). (**b**) Average hydrodynamic diameter of PAsp-g-C18 nanoparticles as a function of DS, showing a decreasing trend at low DS followed by an increase at high DS.

PAsp-g-C18 copolymers with varying DS were self-assembled in aqueous solution via sonication followed by dialysis. The resulting nanostructures were characterized by dynamic light scattering (DLS), which revealed trends in hydrodynamic diameter across the different DS values (Figure 2). In general, the average size of the self-assemblies decreased with increasing DS of hydrophobic grafts, consistent with previous reports [10]. This size reduction is attributed to the increased packing efficiency of hydrophobic chains and decreased hydration of the core-forming segment, leading to more compact structures.

For instance, PAsp-g-C18-1 and PAsp-g-C18-2, with DS values of 4.5 and 9.1 mol%, respectively, formed relatively small spherical assemblies. As the DS increased further (PAsp-g-C18-3, DS 19.0 mol%), the particle size continued to decrease slightly, from 145 ± 1 nm to 130 ± 1 nm. However, a reversal in this trend was observed at higher DS values (PAsp-g-C18-4 and PAsp-g-C18-5 with DS values of 25.7 and 37.6 mol%), where a notable increase in hydrodynamic diameter to approximately 1100 nm was recorded.

**Figure 3:**
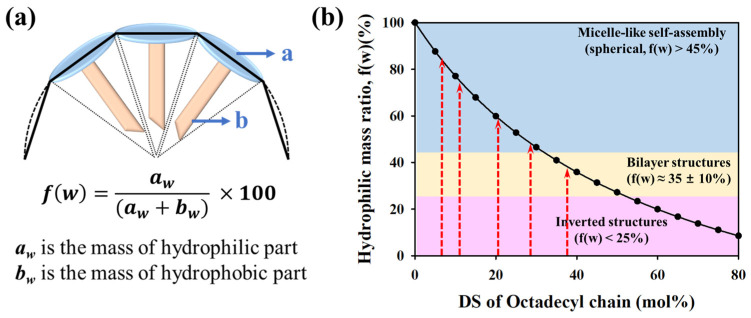
Estimation of the hydrophilic mass fraction (*f*(*w*)) for PAsp-g-C18 self-assembled nanoparticles. (**a**) Schematic illustration for calculating *f*(*w*) in a graft copolymer system, where a denotes the hydrophilic backbone unit (succinimide) and b denotes the hydrophobic octadecyl graft. (**b**) Estimated *f*(*w*) values for PAsp-g-C18 with varying degrees of substitution. The solid line represents the theoretical correlation between *f*(*w*) and the expected self-assembled morphologies.

This size increase at high DS can be rationalized by considering the hydrophilic mass fraction (*f*(*w*)), which represents the mass ratio of hydrophilic to hydrophobic segments. The self-assembled morphology of amphiphilic copolymers is strongly influenced by *f*(*w*), with lower *f*(*w*) values (i.e., more hydrophobic content) favoring the formation of larger structures such as vesicles or lamellae (Figure 3). In the synthesized PAsp-g-C18, *f*(*w*) was calculated as the mass ratio of the succinimide unit in the hydrophilic backbone to the octadecyl graft in the hydrophobic part (Figure 3a). The calculated hydrophilic ratio allowed us to assess the morphological changes according to DS, and the increase in particle size observed at higher DS values appears to be attributable to morphological changes caused by excessive hydrophobic grafting (Figure 3b).

**Figure 4:**
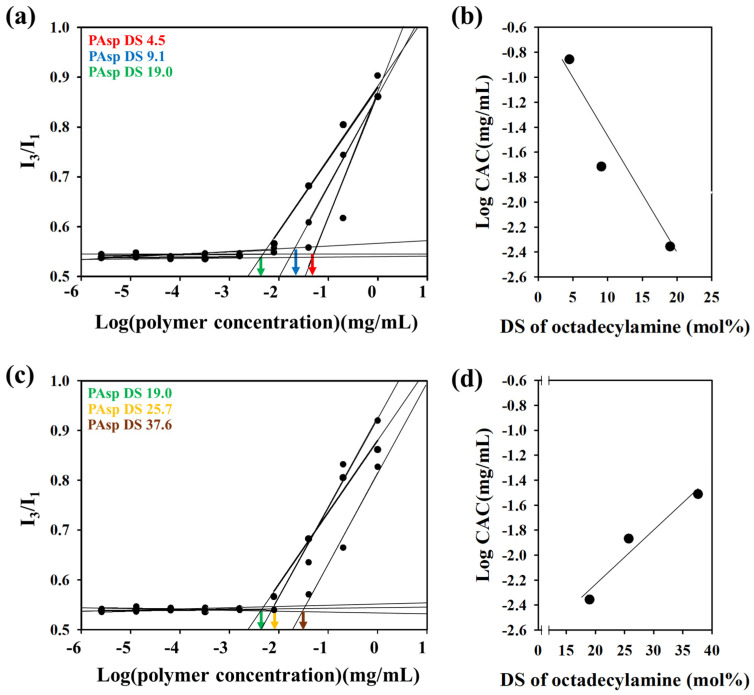
Critical aggregation concentration (CAC) of PAsp-g-C18 copolymers in deionized water determined using pyrene fluorescence. (**a**) Plots of the I_3_/I_1_ fluorescence intensity ratio of pyrene versus the logarithm of PAsp-g-C18 concentrations at DS values of 4.5, 9.1, and 19.0 mol%. (**b**) Linear regression analysis showing a decreasing trend in CAC with increasing DS (4.5–19.0 mol%). (**c**) Plots of I_3_/I_1_ versus the logarithm of PAsp-g-C18 concentrations at higher DS values of 19.0, 25.7, and 37.6 mol%. (**d**) Linear regression showing an increasing trend in CAC at DS values beyond 19.0 mol%, suggesting a morphological transition. Arrows indicate the CAC points (mg/mL) for each sample. Arrows in (**a**,**c**) indicate the CAC values used for the plots in (**b**,**d**).

To further investigate the self-assembly behavior, the critical aggregation concentration (CAC) of each PAsp-g-C18 variant was measured using the fluorescence probe pyrene. The I_3_/I_1_ ratio in pyrene emission spectra was monitored as polymer concentration increased (Figure 4 and Figure S6). For samples with low DS values (4.5, 9.1, and 19.0 mol%), a clear logarithmic decrease in CAC was observed with increasing DS, indicating enhanced hydrophobic interactions that promote aggregation at lower concentrations. This trend is consistent with prior studies on amphiphilic polymer assemblies [11].

Interestingly, this trend did not persist at higher DS values. For PAsp-g-C18-4 and PAsp-g-C18-5 (DS 25.7 and 37.6 mol%), a marked increase in CAC was observed (Figure 4c,d). This unexpected reversal suggests that beyond a certain DS threshold, excessive hydrophobic substitution disrupts the optimal packing of polymer chains, possibly due to steric hindrance and reduced chain flexibility. Similar observations have been reported for graft copolymers, where increased grafting density leads to greater steric hindrance between side chains, limiting efficient chain packing and increasing backbone rigidity while reducing chain flexibility [22,23,24]. These structural constraints may prevent the formation of stable micellar cores and instead favor looser, less efficiently packed assemblies, such as bilayers or vesicles.

Taken together, these results indicate that a critical DS threshold exists (around 19.0 mol%) beyond which self-assembly behavior undergoes a morphological transition (Table 1). This transition corresponds with a shift in hydrophilic/hydrophobic balance, resulting in significant changes in both nanoparticle size and aggregation behavior, as supported by previous literature [25].

### 3.3. Morphological Analysis of PAsp-g-C18 Self-Assembled Nanoparticles

**Figure 5:**
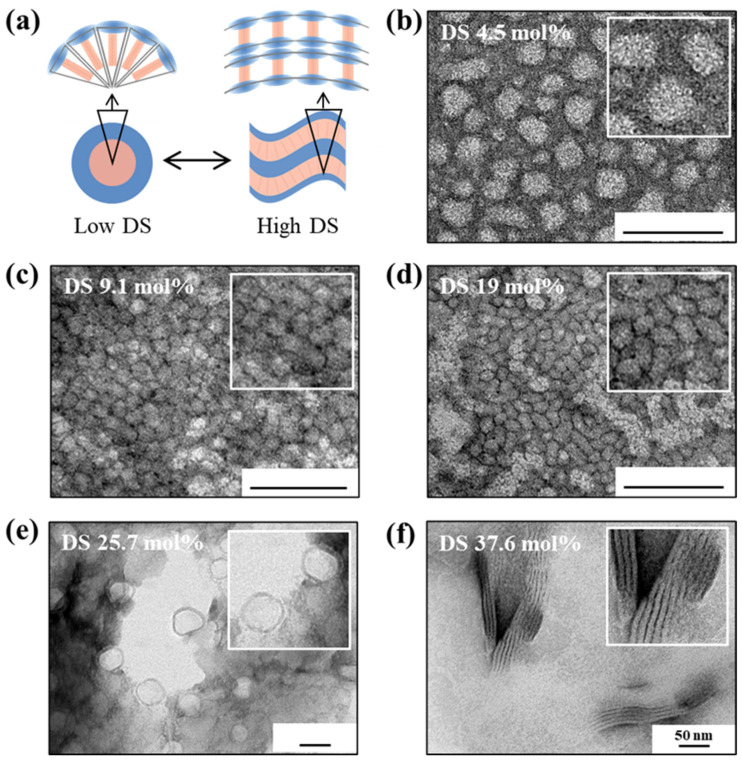
Morphological transition of PAsp-g-C18 self-assembled nanoparticles as a function of DS. (**a**) Schematic illustration of the morphological evolution from spherical micelles to vesicles and lamellar structures with increasing DS. (**b**–**f**) TEM images of PAsp-g-C18 self-assemblies with DS values of 4.5, 9.1, 19.0, 25.7, and 37.6 mol%, respectively. Spherical micelles were observed at low DS, ellipsoidal (rod-like) micelles at DS 19.0, and vesicular and lamellar structures at DS 25.7 and 37.6 mol%, respectively. Scale bar = 50 nm.

Transmission electron microscopy (TEM) images (Figure 5) clearly demonstrate that the morphology of PAsp-g-C18 self-assemblies undergoes distinct transitions as the DS increases. Figure 5a schematically illustrates the conceptual progression of structural changes in the self-assemblies as a function of DS. These morphological transitions were confirmed by direct imaging, revealing a continuum from simple micelles to more complex nanostructures.

At low DS values (4.5 and 9.1 mol%), spherical micelles were predominantly observed. Notably, the particle size at DS 9.1 (7.6 ± 0.9 nm) was smaller than that at DS 4.5 (13.2 ± 2.3 nm), likely due to enhanced hydrophobic interactions promoting tighter packing. These results suggest that at low hydrophobic content, the grafted octadecyl chains provide insufficient driving force for the formation of higher-order assemblies, thereby favoring classical spherical morphologies.

As the DS increased to 19.0 mol%, a clear transition to cylindrical (rod-like) micelles was observed. This morphological shift indicates stronger hydrophobic interactions and improved molecular packing, which promote anisotropic growth. The appearance of elongated structures suggests that polymer chains adopt a more extended conformation, facilitating the formation of rod-like aggregates with increased aspect ratios [26].

At higher DS values (25.7 and 37.6 mol%), the self-assemblies further evolved into vesicular and lamellar morphologies, with sizes increasing to 40.9 ± 6.7 nm and 166.7 ± 40.2 nm, respectively. Specifically, PAsp-g-C18 with DS 25.7 formed bilayer vesicles, while DS 37.6 yielded stacked lamellar structures. The emergence of lamellae at high DS is attributed to the overwhelming hydrophobic contribution, which destabilizes conventional micellar organization and instead favors two-dimensional sheet-like stacking of bilayer membranes [27].

These structural transitions are consistent with established principles of amphiphilic self-assembly, where decreasing hydrophilic mass fraction leads to morphologies with lower curvature. The observed trend aligns with previous studies indicating that as the hydrophilic/hydrophobic balance shifts, polymers tend to form more ordered and extended supramolecular architectures [28,29]. Such morphological control is critical for optimizing drug loading, release kinetics, and cellular uptake, offering valuable insights for the rational design of nanoparticle-based drug delivery systems [30,31].

### 3.4. Cellular Uptake of the Biomimetic Self-Assemblies into the Dermal Cells

**Figure 6:**
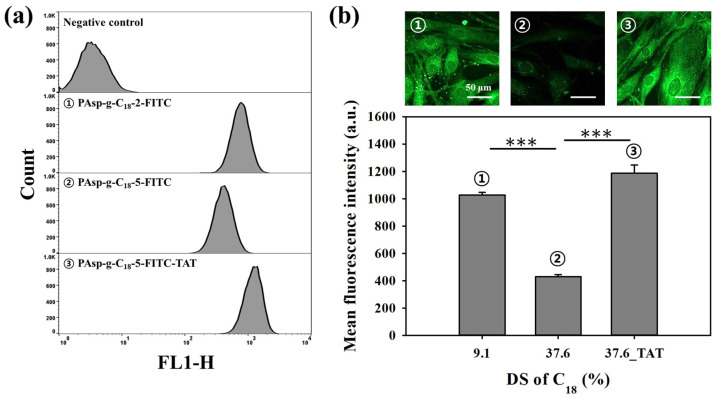
Cellular uptake efficiency of PAsp-g-C18 self-assemblies as a function of grafting mol% of C18 and TAT conjugation. To assess cellular uptake, FITC-labeled PAsp-g-C18 self-assemblies were prepared and incubated with HDFn cells at 37 °C for 2 h (n = 3). (**a**) Flow cytometry analysis comparing uptake levels of spherical (PAsp-g-C18-2-FITC), lamellar (PAsp-g-C18-5-FITC), and TAT-modified lamellar (PAsp-g-C18-5-FITC-TAT) assemblies. (**b**) Representative confocal fluorescence images and corresponding quantification of mean fluorescence intensity in HDFn cells following internalization of the self-assemblies (n = 5, *** *p* < 0.001). Scale bar = 50 μm.

To evaluate the potential of lamellar-structured self-assemblies for drug delivery applications, we compared their cellular uptake with that of conventional spherical micelles using confocal microscopy and flow cytometry (Figure 6). Specifically, PAsp-g-C18-2 (DS 9.1 mol%) was selected as the spherical micelle, while PAsp-g-C18-5 (DS 37.6 mol%) represented the lamellar structure. For visualization, modular self-assemblies were prepared by mixing PHEA-g-C_18_-FITC, PAsp-g-C18-TAT, and PAsp-g-C18 at a ratio of 1:1:8. The DS of C18 in both the FITC-labeled and TAT-conjugated polymers was fixed at 4.5 mol% to minimize variable effects from labeling components.

Human dermal fibroblasts (HDFns) were treated with each formulation, and uptake was assessed 10 min and 2 h post incubation. Confocal microscopy revealed an increase in intracellular fluorescence over time, with stronger signals observed at 2 h (Figure S7). Flow cytometry was subsequently performed for quantitative evaluation following 2 h treatment. When comparing non-TAT-modified self-assemblies, PAsp-g-C18-2-FITC (DS 9.1 mol%) exhibited significantly higher cellular uptake than PAsp-g-C18-5-FITC (DS 37.6 mol%). Both flow cytometry and confocal images demonstrated approximately a 2.0-fold increase in mean fluorescence intensity for PAsp-g-C18-2-FITC compared to PAsp-g-C18-5-FITC. This difference is attributed to the smaller size and spherical morphology of the lower DS formulation, which facilitates more efficient endocytic internalization relative to the larger, more rigid lamellar structures.

To enhance the uptake efficiency of lamellar-type assemblies, we introduced the cell-penetrating peptide TAT to PAsp-g-C18-5-FITC. The resulting PAsp-g-C18-5-FITC-TAT formulation exhibited a substantial increase in cellular internalization. Flow cytometry analysis revealed a pronounced rightward shift in fluorescence intensity distribution compared to the unmodified counterpart (Figure 6a), and confocal images confirmed stronger intracellular fluorescence. Quantitative flow cytometry results showed that the mean fluorescence intensity of PAsp-g-C18-5-FITC-TAT was approximately 1.5-fold higher than that of PAsp-g-C18-2-FITC and 2.6-fold higher than that of PAsp-g-C18-5-FITC. These enhancements were statistically significant (*** *p* < 0.001), demonstrating that the introduction of TAT effectively overcomes the limited uptake associated with lamellar structures.

Collectively, these findings suggest that although high-DS lamellar assemblies exhibit reduced intrinsic cellular uptake, functionalization with CPPs such as TAT can substantially enhance their internalization efficiency, thus broadening their applicability for intracellular drug delivery [32].

## 4. Conclusions

In this study, we successfully synthesized a series of amphiphilic poly(aspartic acid) (PAsp) graft copolymers with varying degrees of octadecyl (C18) substitution and systematically investigated their self-assembly behaviors and cellular delivery potential. The DS significantly influenced the resulting nanostructures, inducing a morphological transition from spherical micelles to rod-like, vesicular, and ultimately lamellar assemblies, as confirmed by TEM analysis. Cellular uptake studies using HDFn cells revealed that spherical self-assemblies with lower DS (e.g., DS 9.1) exhibited substantially higher uptake efficiency compared to lamellar structures formed at higher DS (e.g., DS 37.6), likely due to differences in particle size and morphology affecting endocytic internalization. To overcome the inherently low uptake efficiency of the lamellar structures, we introduced the cell-penetrating peptide (TAT) into the lamellar-forming copolymer (PAsp-g-C18-5). This modification significantly enhanced cellular internalization without inducing cytotoxicity. Collectively, our findings demonstrate that both structural tuning and surface functionalization are critical strategies for optimizing the cellular delivery performance of self-assembled graft copolymers. In particular, the ability to modulate nanostructure morphology through hydrophobic substitution, combined with bioactive ligand conjugation, offers a versatile and effective approach for improving the intracellular delivery of nanocarriers. This strategy holds strong potential for the rational design of advanced polymer-based drug delivery systems and could be applied in areas such as targeted cancer therapy, regenerative medicine, and gene delivery.

## Figures and Tables

**Table 1:** Molecular characterization of poly(aspartic acid)-g-C18 series.

Polymers	Feed ^a^	DS ^b^	Number ^c^	Morphology ^d^	*f*(*w*) ^e^	CAC ^f^
PAsp	100/0	-	-	-	-	
PAsp-C18-1	95/5	4.5	26	Spherical	88.8	5.1 × 10^−2^
PAsp-C18-2	90/10	9.1	53	Spherical	78.9	1.9 × 10^−2^
PAsp-C18-3	80/20	19.0	111	Ellipsoidal	61.4	4.4 × 10^−3^
PAsp-C18-4	60/40	25.7	151	Vesicle	51.9	7.5 × 10^−3^
PAsp-C18-5	40/60	37.6	221	Lamellar	38.3	3.1 × 10^−2^

^a^ Feed mole ratio of Poly(succinimide) unit/Octadecylamine. ^b^ Degree of substitution (succinimide units amine groups, mol%) determined based on ^1^H-NMR of graft copolymers. ^c^ Number of octadecylamine per one polymer chain. ^d^ Morphology of self-assembled nanoparticle obtained by TEM images. ^e^ Estimation of the *f*(*w*) of octadecylamine-conjugated PAsp. ^f^ Critical aggregation concentration (mg/mL) determined by pyrene assay.

## Data Availability

The original contributions presented in this study are included in the article/Supplementary Materials. Further inquiries can be directed to the corresponding author.

## Supplementary Materials

The following supporting information is available online at: https://www.mdpi.com/article/10.3390/polym17172373/s1. Figure S1: Synthetic scheme of PAsp-g-TAT-C18. Figure S2: Synthetic scheme of PHEA-g-C18-NH_2_. Figure S3: ^1^H NMR spectra of (a) PSI, (b) PSI-g-C18 (DS 4.5), (c) PSI-g-C18 (DS 9.1), (d) PSI-g-C18 (DS 19.0), (e) PSI-g-C18 (DS 25.7), and (f) PSI-g-C18 (DS 37.6). Figure S4: Fluorescence emission spectra of pyrene in polymer solutions at various concentrations: (a) PSI-g-C18-1 (DS 4.5), (b) PSI-g-C18-2 (DS 9.1), (c) PSI-g-C18-3 (DS 19.0), (d) PSI-g-C18-4 (DS 25.7), and (e) PSI-g-C18-5 (DS 37.6). Figure S5: Confocal microscopy images of HDFn cells incubated with PAsp-g-C18 self-assemblies for 10 min (left) and 2 h (right) at 37 °C: (a) DS 9.1 with TAT, (b) DS 25.7 with TAT, (c) DS 37.6 with TAT, and (d) DS 37.6 without TAT. Figure S6: Fluorescence emission spectra of pyrene in polymer solutions at various concentrations: (a) PAsp-g-C18-1 (DS 4.5), (b) PAsp-g-C18-2 (DS 9.1), (c) PAsp-g-C18-3 (DS 19.0), (d) PAsp-g-C18-4 (DS 25.7), and (e) PAsp-g-C18-5 (DS 37.6). Figure S7: Confocal microscopy images of HDFn cells incubated with PAsp-g-C18 self-assemblies for 10 min (left) and 2 h (right) at 37 °C: (a) DS 9.1 with TAT, (b) DS 25.7 with TAT, (c) DS 37.6 with TAT, and (d) DS 37.6 without TAT.
