# Supplementary material for: Tuning the Structure of Poly(aspartic acid)s’ Self-Assemblies to Enhance Cellular Uptake
Source: Polymers (Basel). 2025 Aug 31;17(17):2373. doi: 10.3390/polym17172373 (PMC12431294; doi:10.3390/polym17172373)
Supplement: Supplementary file 1 [file polymers-17-02373-s001.zip › polymers-3798709-supplementary.pdf]

Tuning the Structure of Poly(aspartic acid)s' Self-Assemblies to Enhance Cellular Uptake

Jimin Jeong †, Junwoo Lim †, Sungwoo Cho, Sa Ra Han, Suk Hyeon Hong and Jae Hyun Jeong *

Department of Chemical Engineering, Soongsil University, 369, Sangdo-Ro, Dongjak-Gu, Seoul 06978, Republic of Korea

* Correspondence: nfejjh@ssu.ac.kr; Tel.: +82-2-828-7043; Fax: +82-2-812-5378

† These authors contributed equally to this work.

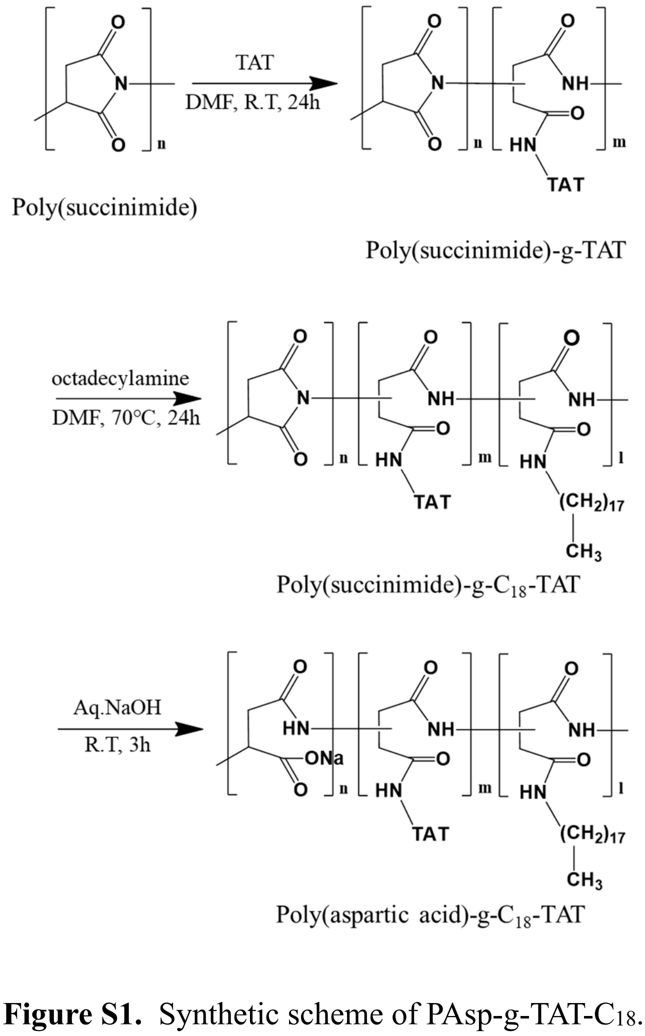

Figure S1. Synthetic scheme of PAsp-g-TAT-C_{18}.

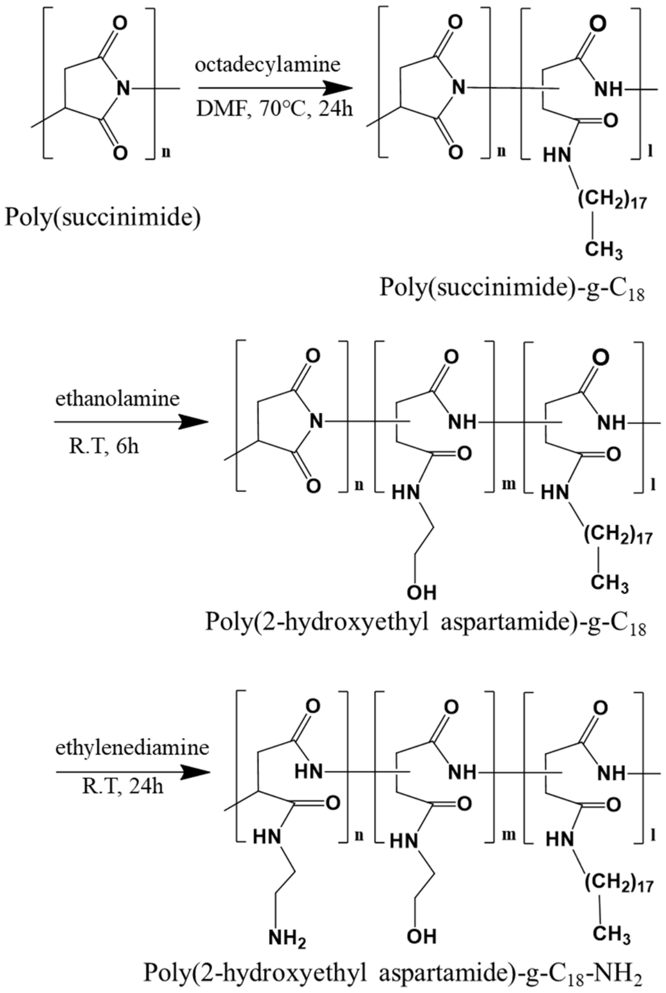

Figure S2. Synthetic scheme of PHEA-g-C18-NH_2.

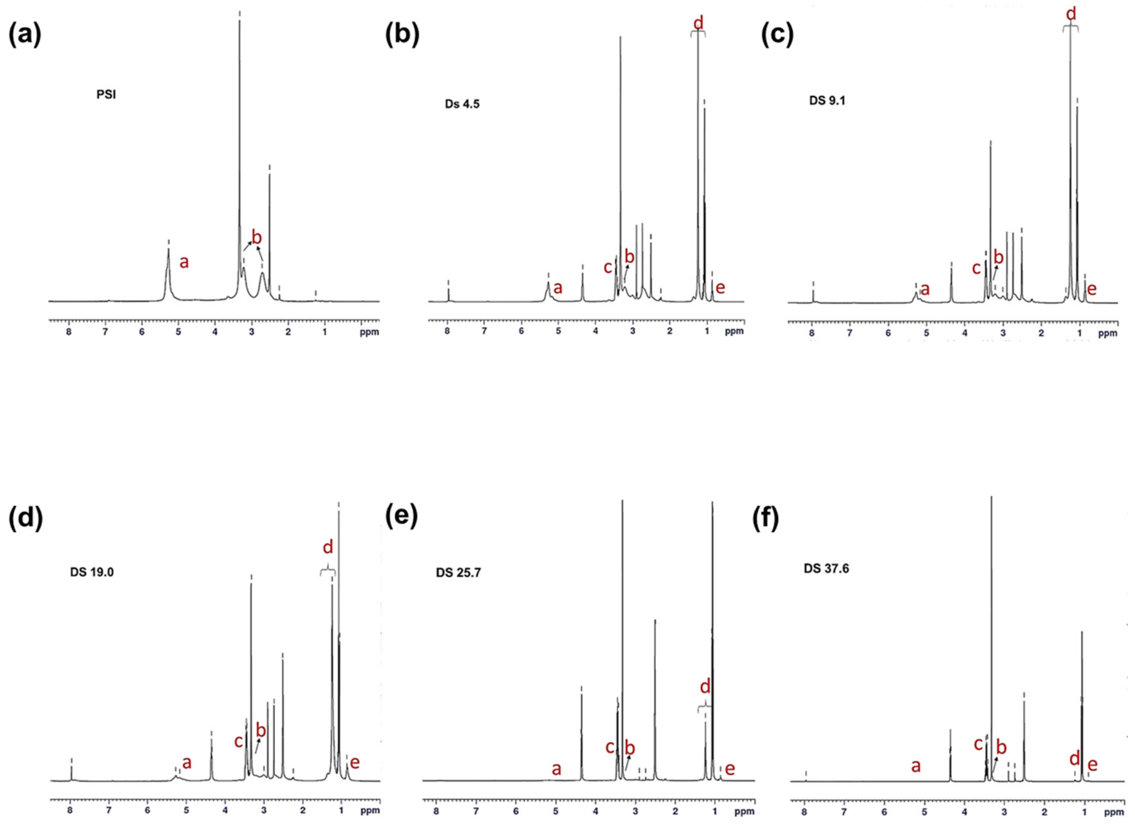

Figure S3. ^{1}H NMR spectra of (a) PSI, (b) PSI-g-C18 (DS 4.5), (c) PSI-g-C18 (DS 9.1), (d) PSI-g-C18 (DS 19.0), (e) PSI-g-C18 (DS 25.7), and (f) PSI-g-C18 (DS 37.6).

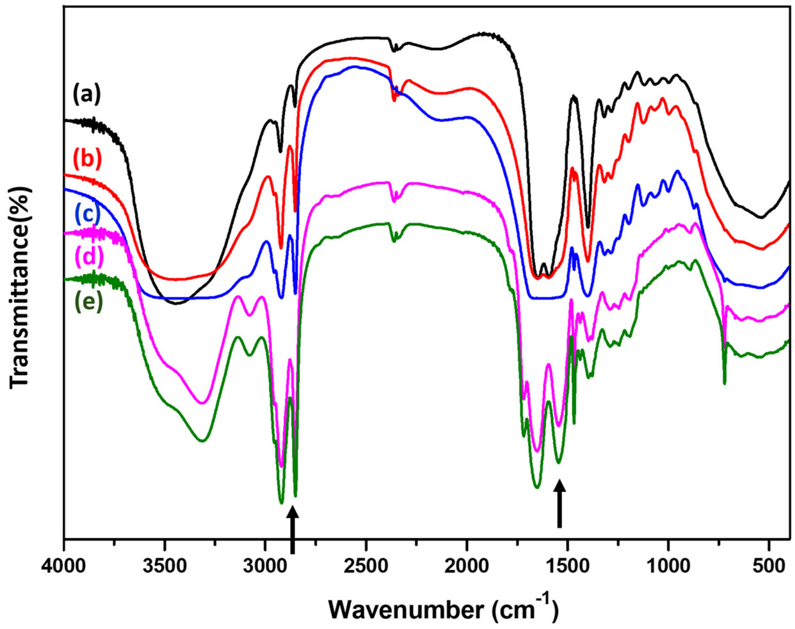

Figure S4. FT-IR spectra of (a) PAsp-g-C18 (DS 4.5), (c) PAsp-g-C18 (DS 9.1), (d) PAsp-g-C18 (DS 19.0), (e) PAsp-g-C18 (DS 25.7), and (f) PAsp-g-C18 (DS 37.6). The arrows mark the characteristic C–H stretching region (2850–3000 cm^{-1}) and the amide-related bands (1550–1650 cm^{-1}).

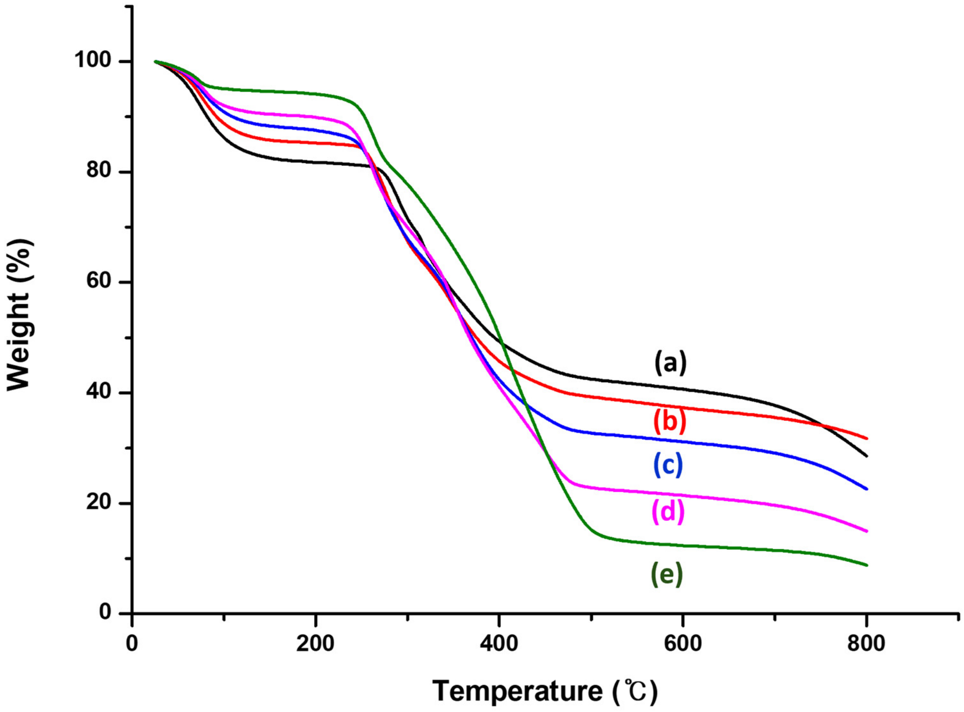

Figure S5. TGA curves of (a) PAsp-g-C18 (DS 4.5), (c) PAsp-g-C18 (DS 9.1), (d) PAsp-g-C18 (DS 19.0), (e) PAsp-g-C18 (DS 25.7), and (f) PAsp-g-C18 (DS 37.6).

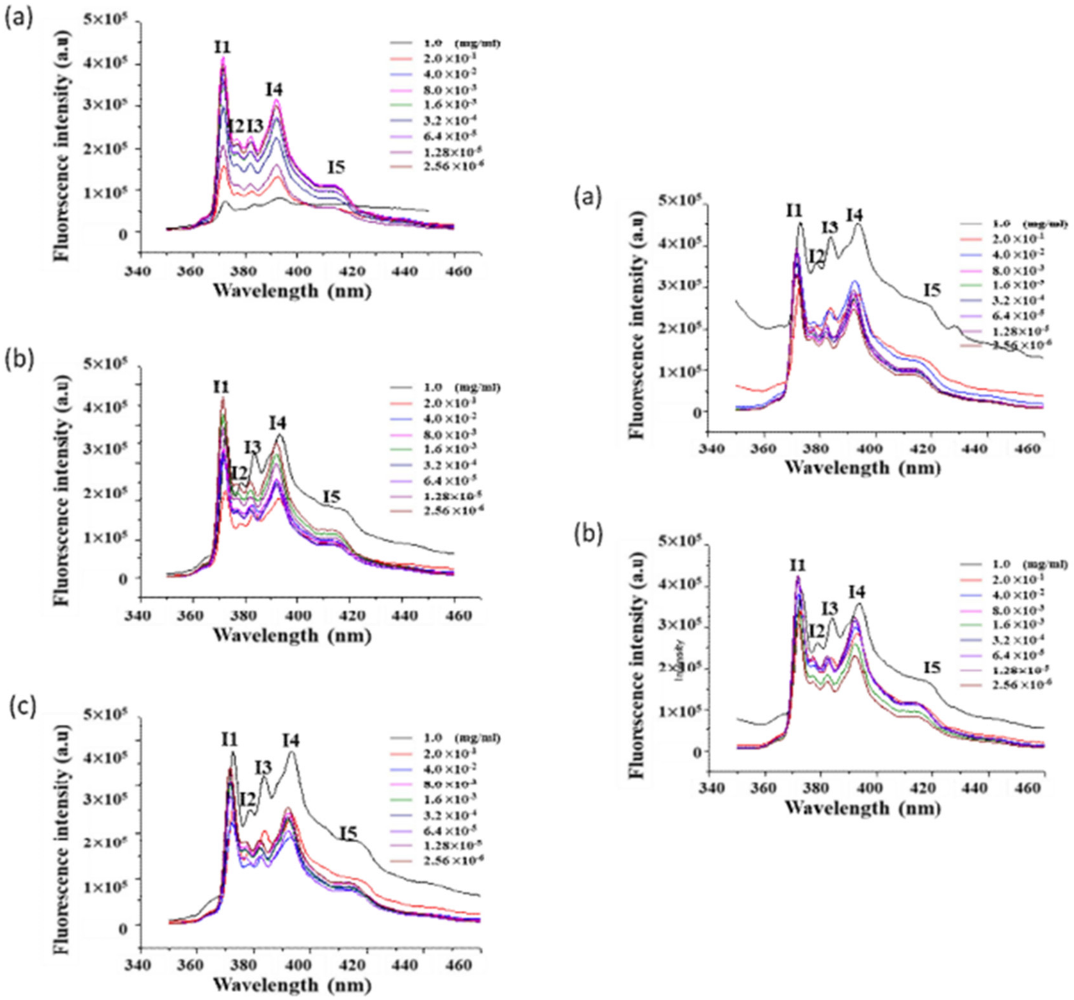

Figure S6. Fluorescence emission spectra of pyrene in polymer solutions at various concentrations: (a) PAsp-g-C18-1 (DS 4.5), (b) PAsp-g-C18-2 (DS 9.1), (c) PAsp-g-C18-3 (DS 19.0), (d) PAsp-g-C18-4 (DS 25.7), and (e) PAsp-g-C18-5 (DS 37.6).

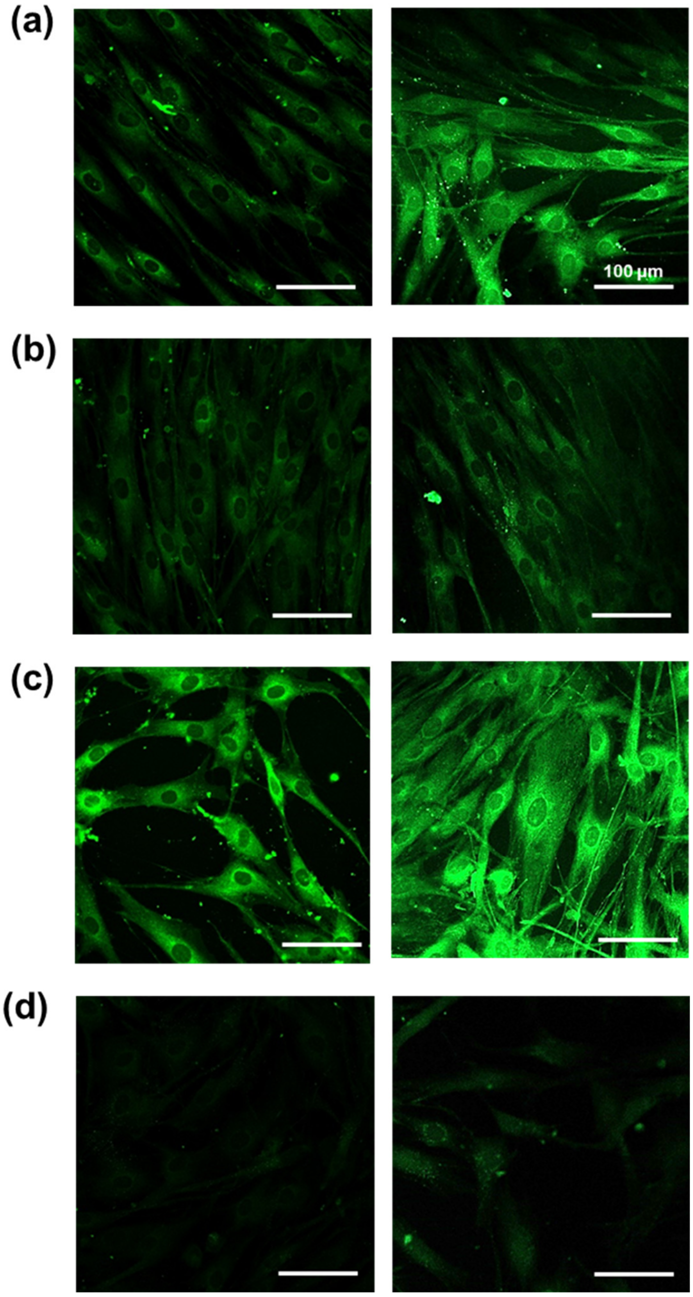

Figure S7. Confocal microscopy images of HDFn cells incubated with PAsp-g-C18 self-assemblies for 10 min (left) and 2 h (right) at 37 °C: (a) DS 9.1 with TAT, (b) DS 25.7 with TAT, (c) DS 37.6 with TAT, and (d) DS 37.6 without TAT.
